# Anxiety and depression among patients with non-tuberculous mycobacterial disease in Shanghai: a cross-sectional study

**DOI:** 10.3389/fpsyt.2023.1132675

**Published:** 2023-05-22

**Authors:** Sikang Ni, Yuting Chen, Bijie Hu, Zheng Yuan

**Affiliations:** Zhongshan Hospital, Fudan University, Shanghai, China

**Keywords:** non-tuberculous mycobacterial disease, anxiety, depression, Department of Infection, psychology

## Abstract

**Objective:**

To understand the mental health status and its influencing factors among patients with non-tuberculous mycobacterial disease and to provide a reference for medical staff to formulate scientific and feasible intervention strategies.

**Methods:**

A total of 114 patients diagnosed with non-tuberculous mycobacillosis during hospitalization in the Department of Infection from September 2020 to April 2021 were selected as the research participants. Participants’ mental health status and related factors were evaluated using a self-made general patient information questionnaire, self-rating Anxiety Scale (SAS), and self-rating Depression Scale (SDS).

**Results:**

Among 114 patients with non-tuberculous mycosis, 61 (53.51%) exhibited depressive symptoms, and the SDS score was 51.15 ± 13.04, which was higher than the national norm of 41.88 ± 10.57 (*p* < 0.05); further, 39 patients (34.21%) showed anxiety symptoms, and the SAS score was 45.75 ± 10.81, which was significantly higher than the national norm of 29.78 ± 10.07 (*p* < 0.05). Body mass index and monthly household income had significant effects on depression in patients with non-tuberculous mycobacterial disease (*p* < 0.05). Educational level had a significant effect on the anxiety state of patients with non-tuberculous mycobacterial disease (*p* < 0.05).

**Conclusion:**

Patients with non-tuberculous mycobacterial disease are prone to depression and anxiety. Nurses should pay attention to it in clinical work for the timely identification of and intervention for anxiety and depression and intervene.

## 1. Introduction

With the development of social economy and the transformation of modern medical models, emotional problems, such as anxiety and depression, are gradually becoming a concern. The treatment not only focuses on the physical health of patients but also their mental health. Previous studies have found that the occurrence and development of chronic diseases are closely related to psychological problems, such as anxiety and depression (1). Non-tuberculous mycobacterial (NTM) disease refers to diseases of related tissues or organs caused by human infection with non-tuberculous mycobacteria. Owing to the long course of the disease, complex treatment, numerous adverse reactions, and poor public awareness of relevant knowledge, multiple factors can easily cause patients to experience negative emotions such as anxiety and depression during treatment (2). Furthermore, patients with NTM disease show more typical personality characteristics, like sensitive and anxious; this is also known as “Mrs. Windermere syndrome” (3). At present, the current situation of anxiety and depression in patients with non-tuberculous mycobacterial disease is not optimistic. According to statistics, about 30% of non-tuberculous mycobacterial disease patients are accompanied by emotional problems such as anxiety and depression, and these patients often feel anxiety, depression, loss and other emotions, which seriously affect the quality of life of patients. The causes of anxiety and depression in patients with nontuberculous mycobacterial disease are complex, which may be related to various factors such as the patient’s physical condition, psychological state, and social environment. For example, patients may feel anxious and depressed because of physical discomfort, and may also feel anxious and depressed because of worries about the disease. In addition, changes in the social environment may also have an impact on the patient’s emotions, such as work pressure, family conflicts, etc. In China, the psychological characteristics of patients with NTM disease have not been sufficiently examined. SAS and SDS are commonly used as anxiety and depression assessment tools to assess the degree of anxiety and depression in patients. Therefore, this study aimed to assess the anxiety and depression levels of patients with non-tuberculous mycobacterial disease, explore the influencing factors, and provide a reference for medical staff to formulate scientific and feasible intervention strategies.

## 2. Materials and methods

### 2.1. Clinical data

**Figure 1:**
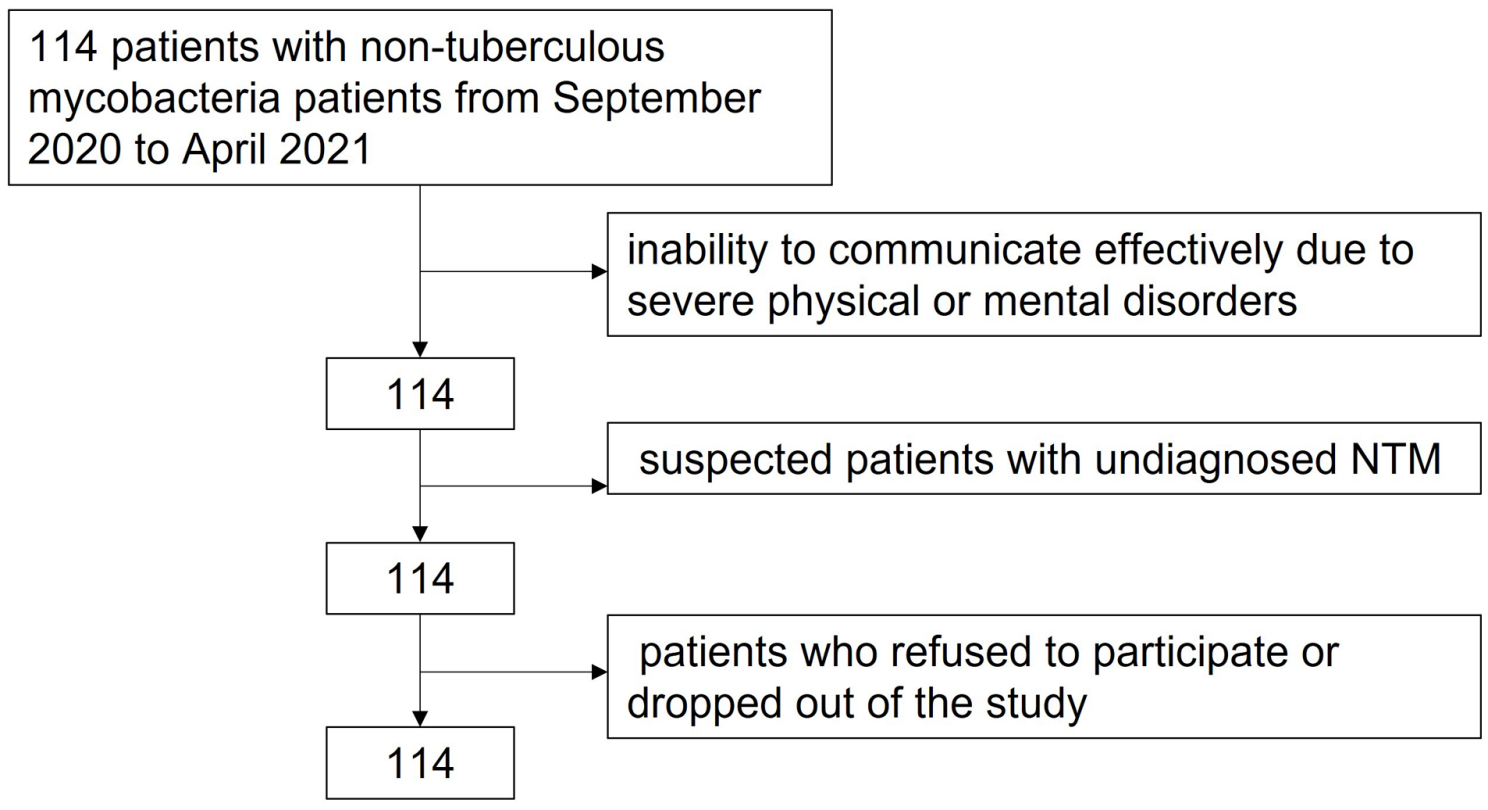
The flow chart of the study group.

From September 2020 to April 2021, 114 patients with non-tuberculous mycobacteria were diagnosed during hospitalization in the Department of Infection. The patient health status questionnaire, self-rating anxiety scale (SAS), and self-rating depression scale (SDS) were used to evaluate patients’ mental health status and related factors. The inclusion criteria were as follows: (1) patients diagnosed with NTM disease according to the 2020 version of the guidelines for the diagnosis and treatment of the disease (4), (2) patients with no organic brain disease or mental illness history, and (3) patients with the ability to communicate with others without barriers and willingness to participate in this study. The exclusion criteria were as follows: (1) inability to communicate effectively due to severe physical or mental disorders, (2) suspected patients with undiagnosed NTM, and (3) patients who refused to participate or dropped out of the study. Our study was approved by the ethical committee of Ethics Committee of Zhongshan Hospital Fudan University (Approval No: B2020-411R) (Figure 1).

### 2.2. Survey tools

#### 2.2.1. General information questionnaire

A self-made general information questionnaire was used to collect data on patients’ sex, age, height, weight, education level, marital status, monthly household income, medical expense payment method, monthly treatment cost, chronic diseases, and other information.

#### 2.2.2. Self-rating depression scale

SDS consists of 20 items and four dimensions: mental affective symptoms (items 1 and 3), somatic disorders (items 2, 4, 5, 6, 7, 8, 9, and 10), psychomotor disorders (items 12 and 13), and depressive psychological disorders (items 11, 14, 15, 16, 17, 18, 19, 20). The scores of the 20 items were multiplied by 1.25, and the integer was used to obtain the scores of the SDS. Higher scores indicate more severe depression. The score standards were as follows: 53–62 points for mild depression, 63–72 points for moderate depression, and ≥ 73 points for severe depression. The reliability coefficient of the scale was 0.92, and its validity coefficient was 0.842 (5–9).

#### 2.2.3. Self-rating anxiety scale

SAS includes 20 items and four dimensions, namely anxiety mood (items 1, 2, 3, and 4), autonomic nervous dysfunction (items 7, 8, 10, 11, 12, 14, 15, and 18), exercise stress (items 6, 9, 13, 17, 19, and 20), and mixed symptoms of anxiety mood and autonomic nervous function (items 5 and 16). The scores of the 20 items were multiplied by 1.25 and the integer was used to obtain the score of the Self-rating Anxiety Scale. Higher scores indicate more severe anxiety. The score standards were as follows: 50–59, mild anxiety; 60–69, moderate anxiety; and ≥70, severe anxiety. The reliability coefficient of the scale was 0.931, and the validity coefficient was 0.810 (5–9).

### 2.3. Survey methods

When a nurse admits a patient diagnosed with NTM, she participates in the study with her consent, after filling out the informed consent form. Nurse administered the questionnaires to patients diagnosed with NTM disease during admission in a quiet, undisturbed environment. If it was difficult for the interviewee to fill out the questionnaires on their own, the nurse assisted them by reading out the questions and noting the answers. After the completion of the questionnaires, a total of 114 valid responses were obtained, with an effective recovery rate of 100%.

### 2.4. Statistical analysis

Microsoft Excel was used for data entry and leak detection. The mean and standard deviation were used to describe the overall scores and scores of each dimension of the SAS and SDS scales, t-test and ANOVA were used to compare the depression and anxiety of NTM patients with different characteristics, Subsequently, variables with a *p* value <0.05 were included in the multivariate linear regression was used to analyze the influencing factors of depression and anxiety in NTM patients. All analyses were performed by using SPSS version 25.0 (IBM SPSS Statistics, IBM Corporation). *p* < 0.05 was considered statistically significant.

## 3. Results

### 3.1. Depression and anxiety in patients with NTM disease

#### 3.1.1. Depression and anxiety overall scores in patients with NTM disease

Among the 114 patients with NTM, the mean SDS score was 51.15 ± 13.04, higher than the national norm of 41.88 ± 10.57 (10), and the difference was statistically significant (*p* < 0.05). The mean SAS score was 45.75 ± 10.81, which was significantly higher than the national norm of 29.78 ± 10.07 (6), and the difference was statistically significant (*p* < 0.05; Table 1). Overall, 61 patients (53.51%) demonstrated depressive symptoms, and 39 (34.21%) showed anxiety symptoms.

**Table 1:** Comparison of SDS and SAS scores of patients with NTM disease with the national norm (x ± s, points).

Item	SDS		SAS	
	*n*	Score	*n*	Score
The national norm	1,340	41.88 ± 10.57	1,158	29.78 ± 10.07
NTM patients	114	51.15 ± 13.04	114	45.75 ± 10.81
*t*	7.594		15.772	
*p*	0.000		0.000	

**Table 2:** SDS dimension-wise score and sort (x ± s, points).

Dimension	Number of entries	Dimension score	Entries score	Rank
Mental emotional symptoms	2	3.18 ± 1.14	1.59 ± 0.57	4
Somatoform disorders	8	15.89 ± 3.96	1.99 ± 0.49	3
Psychomotor disturbance	2	4.32 ± 1.47	2.11 ± 0.74	2
Depressive psychological disorder	8	17.54 ± 6.15	2.19 ± 0.77	1

#### 3.1.2. Depression and anxiety scores by dimension in patients

SDS dimension-wise score and sort (Table 2),SAS dimension-wise score and sort (Table 3).

### 3.2. Comparison of depression and anxiety in patients with NTM disease with different characteristics

Among the participants, body mass index (BMI) and education level were correlated with depression and anxiety (*p* < 0.05). In addition, monthly household income and payment method of medical expenses were significantly correlated with depression (*p* < 0.05), whereas sex and diabetes were significantly associated with anxiety (*p* < 0.05; Table 4).

**Table 3:** SAS dimension-wise score and sort (x ± s, points).

Dimension	Number of entries	Dimension score	Entries score	Sort
Anxiety mood	4	6.36 ± 2.35	1.59 ± 0.59	3
Autonomic dysfunction	8	12.44 ± 3.81	1.55 ± 0.48	4
Sports tension	6	13.16 ± 3.46	2.19 ± 0.58	2
Mixed symptoms of anxiety and Autonomic nervous function	2	4.65 ± 3.09	2.32 ± 1.55	1

**Table 4:** Comparison of depression and anxiety scores in patients with NTM disease with different characteristics (x ± s, points).

Item		*n*	SDS scores	*t*/*F*	*p*	SAS scores	*t*/*F*	*p*
Sex	Male	51	50.02 ± 14.33	−0.829	0.409	43.50 ± 10.05	−2.207	0.045
	Female	63	52.06 ± 11.92			47.58 ± 11.15		
Age	≤50	48	49.92 ± 12.06	−0.858	0.393	46.04 ± 10.52	0.239	0.812
	>50	66	52.05 ± 13.72			45.55 ± 11.10		
BMI	<18.5	21	57.02 ± 9.88	6.460	0.002	51.31 ± 8.52	6.530	0.002
	18.5–23.9	81	51.16 ± 12.86			45.48 ± 10.62		
	≥24	12	40.83 ± 13.51			37.92 ± 11.12		
Education level	High school or below	69	53.79 ± 11.63	3.782	0.026	48.42 ± 10.77	6.334	0.002
	Junior college	28	47.50 ± 15.89			40.45 ± 9.19		
	Bachelor’s degree and above	17	46.47 ± 11.05			43.68 ± 10.28		
Marital status	Unmarried	16	48.75 ± 12.37	0.374	0.689	45.94 ± 11.18	0.147	0.864
	Married	91	51.41 ± 13.32			45.56 ± 10.94		
	Divorced or widowed	7	53.21 ± 11.61			47.86 ± 9.40		
Monthly household income	<3,000 RMB	31	53.79 ± 11.34	3.151	0.047	47.18 ± 9.52	2.668	0.074
	3,000–6,000 RMB	56	52.23 ± 11.37			46.96 ± 11.28		
	>6,000 RMB	27	45.88 ± 13.12			41.62 ± 10.56		
Medical insurance payment	Without	13	56.35 ± 7.85	2.295	0.031	49.04 ± 9.10	1.164	0.247
	With	101	50.47 ± 13.44			45.33 ± 10.98		
Percentage of monthly treatment costs	<20%	38	49.21 ± 11.33	2.229	0.089	42.83 ± 8.37	2.371	0.074
	20%–40%	39	52.18 ± 12.49			48.56 ± 13.02		
	40%–60%	12	45.00 ± 14.29			42.71 ± 10.21		
	>60%	25	55.45 ± 11.68			47.30 ± 9.65		
Hypertension	With	12	54.38 ± 13.73	0.905	0.367	48.75 ± 16.74	1.014	0.313
	Without	102	50.77 ± 12.97			45.40 ± 9.96		
Diabetes mellitus	With	3	40.83 ± 6.29	−1.395	0.166	32.50 ± 13.07	−2.187	0.031
	Without	111	51.43 ± 13.07			46.11 ± 10.73		
Cardiovascular disease	With	3	41.67 ± 17.25	−1.281	0.203	42.50 ± 13.17	−0.527	0.599
	Without	111	51.40 ± 12.91			45.84 ± 10.80		
Malignancy and blood systemic diseases	With	5	58.75 ± 6.37	1.338	0.184	54.00 ± 10.80	1.759	0.081
	Without	109	50.80 ± 13.17			45.38 ± 10.71		

**Table 5:** Independent variable assignment table.

Variable	Influencing factor	Assignment
*X*1	Gender	Male = 1,female = 0
*X*2	BMI	Set 2 dummy variable
		Dummy variable 1:<18.5 = 0， ≥ 24 = 1,18.5–23.9 = 0 Dummy variable 2:<18.5 = 0， ≥ 24 = 0，18.5–23.9 = 1
*X*3	Education level	Set 2 dummy variable
		Dummy variable 1:High school or below =0，Junior college =1，Bachelor degree and above =0 Dummy variable 2:High school or below =0,Junior college =0，Bachelor degree and above =1
*X*4	Family average income	Set 2 dummy variable
		Dummy variable 1:family average income >6,000 RMB = 0，family average income 3,000–6,000 RMB = 1，family average income <3,000 RMB = 0 Dummy variable 2:family average income >6,000 RMB = 0，family average income 3,000–6,000 RMB = 0，family average income <3,000 RMB = 1
*X*5	Medical payment method	Without medical insurance payment = 0, With medical insurance payment = 1
*X*6	With or without diabetes mellitus	Without diabetes mellitus = 0，With diabetes mellitus = 1

**Table 6:** Results of multivariate analysis of depression in patients with NTM disease.

	Non-standardized coefficient		Standardized coefficient	*t*	*p*	95% confidence interval	
	*B*	standard error	Beta			Lower Bound	Upper Bound
(constant)	49.113	4.966		9.889	0.000	39.267	58.959
BMI ≥ 24	−12.445	3.908	−0.294	−3.184	0.002	−20.193	−4.697
BMI 18.5–23.9	5.390	3.094	0.161	1.742	0.084	−0.744	11.524
Junior college	−2.345	2.948	−0.078	−0.796	0.428	−8.189	3.499
Bachelor’s degree and above	−2.932	4.357	−0.080	−0.673	0.502	−11.570	5.706
Family average income 3,000–6,000 RMB	5.408	3.729	0.208	1.450	0.150	−1.984	12.800
Family average income <3,000 RMB	8.467	4.105	0.290	2.063	0.042	0.328	16.606
With medical insurance payment	−1.795	3.729	−0.044	−0.481	0.631	−9.187	5.598

*R*^2^ = 0.199, *F* = 3.761, *p* = 0.001.

**Table 7:** Results of multivariate analysis of anxiety in patients with NTM disease.

	Non-standardized coefficient		Standardized coefficient	*t*	*p*	95% confidence interval	
	*B*	standard error	Beta			Lower Bound	Upper Bound
(constant)	48.889	1.630		29.989	0.000	45.658	52.121
Male	−2.060	1.980	−0.095	−1.040	0.301	−5.985	1.866
BMI ≥ 24	−5.556	3.289	−0.158	−1.689	0.094	−12.075	0.964
BMI 18.5–23.9	4.726	2.529	0.170	1.869	0.064	−0.287	9.739
Junior college	−5.644	2.328	−0.226	−2.424	0.017	−10.260	−1.028
Bachelor’s degree and above	−5.840	2.719	−0.193	−2.148	0.034	−11.230	−0.451
With diabetes mellitus	−9.125	6.196	−0.136	−1.473	0.144	−21.408	3.157

*R*^2^ = 0.206, *F* = 4.618, *p* = 0.000.

### 3.3. Regression analysis of factors influencing anxiety and depression

Anxiety and depression were used as dependent variables, and sex, BMI, education level, monthly household income payment method of medical expenses, and presence or absence of diabetes were used as independent variables. Multivariate linear regression analysis was performed (Table 5).

#### 3.3.1. Binary logistic regression analysis of factors influencing depression

The depression score was used as the dependent variable, the meaningful variables in the single-factor results were used as the independent variables, and the input method was the linear regression model. The results showed that patients with NTM disease with lower BMI were more likely to experience depressive symptoms than those with higher BMI (*p* < 0.05). Patients with NTM disease with a monthly household ncome of <3,000 yuan were more likely to experience depressive symptoms than those with an income of >6,000 yuan (*p* < 0.05; Table 6).

#### 3.3.2. Binary logistic regression analysis of factors influencing anxiety

The anxiety score was the dependent variable and the single-factor results of meaningful variables were the independent variables, using the linear regression model as an input method. The results showed that participants with a college degree or higher were less likely to experience anxiety than those with a high school degree or lower (*p* < 0.05; Table 7).

## 4. Discussion

Psychological problems have been a global problem, and according to the World Health Organization, more than 350 million people are afflicted by depression worldwide, which has become the fourth most common disease in the world and is still growing rapidly. Who predicts that depression will become the first disease burden worldwide in 2030 (11). In the Chinese Mental Health Survey (CMHS), it was shown that anxiety disorders were the most prevalent class of mental disorders (12), at the same time, many diseases are produced, symptoms, types, development as well as the length of the disease, the outcome and prognosis of the patients are a result of changes in behavioral and emotional aspects induced by psychological and social stressful stimuli, and the psychological conditions after the illness can also continuously affect the condition. In summary, psychological problems are in clinical nursing work that need us to detect and intervene in a timely manner.

Psychological problems has been emphasized in the field of NTM disease (13). According to the existing data, the incidence and prevalence of NTM disease are increasing worldwide (14, 15). Previous studies have also found that people with underlying lung diseases, such as bronchiectasis, pneumoconiosis, TB, etc., or HIV infection, tumors, organ transplantation, and other diseases are more likely to become sick (16–18). As this chronic debilitating disease requiring long-term antibiotic therapy could amplify patients’ negative emotions. So the mental health status of patients with NTM disease are important and deserve our attention. Patients with NTM disease have a long course of the disease. During the course of the disease, they will experience repeated acute exacerbations and be repeatedly hospitalized. Long-term medication is needed. There are inferiority complexes and psychological shadows, indifferent interpersonal relationships, and some even lose the ability to work, increasing their economic burden and leading to depression, anxiety, mania, and sleep disorders (19). Overall, the psychological problems of patients with NTM lung disease are many and complex and deserve social attention. However, In China, the psychological characteristics of patients with NTM disease have not been sufficiently examined.

**Figure 2:**
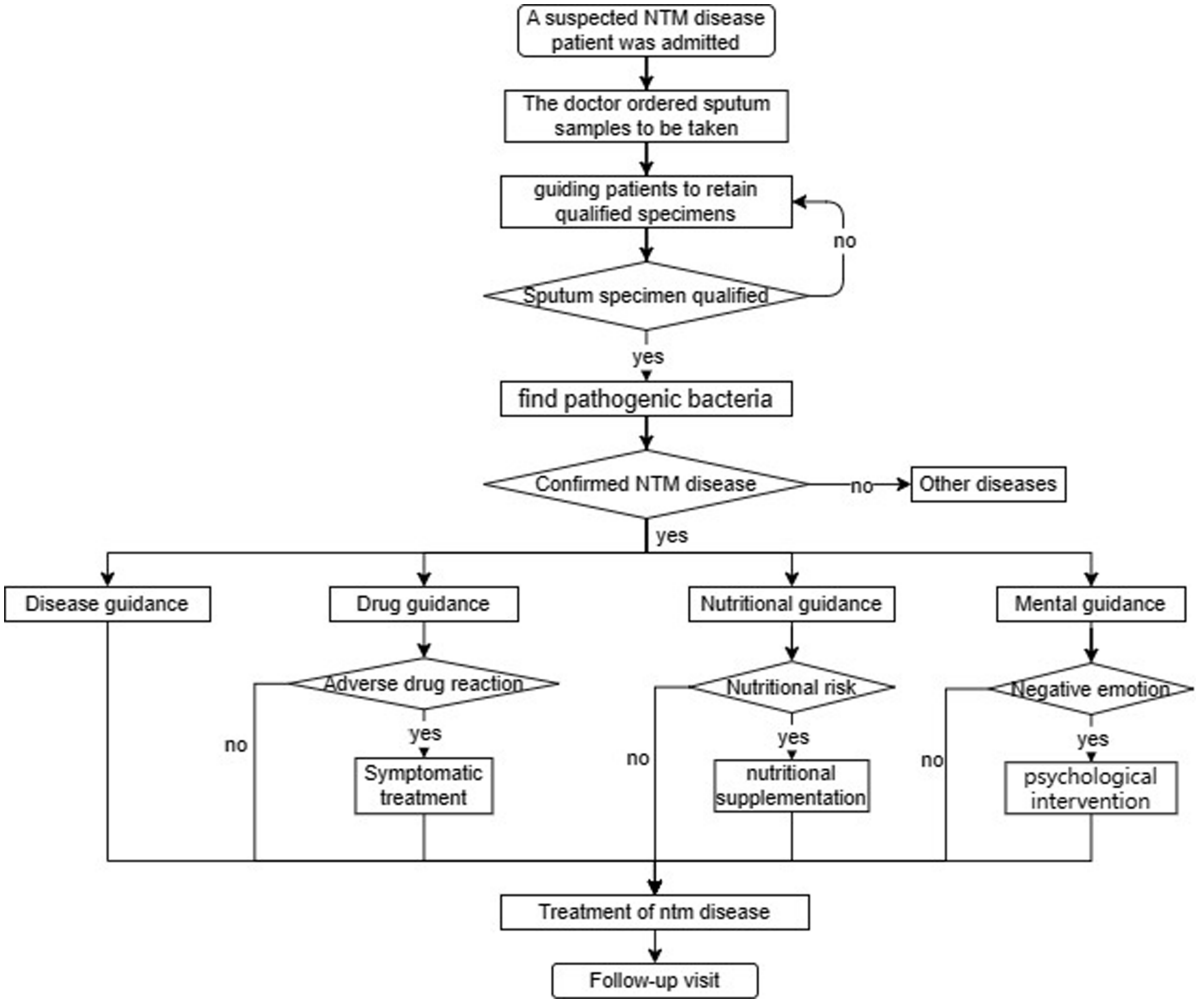
Nurse in the management of the patient diagnosed with NTM.

In this article, we aimed to understand the mental health status and its influencing factors among patients with non-tuberculous mycobacterial disease and to provide a reference for medical staff to formulate scientific and feasible intervention strategies. We found that patients with NTM disease were more prone to depression and anxiety and that their depression and anxiety scores were much higher than those of the general population, Previous study has also found that 22.8% and 22.5% of the patients with NTM disease experienced anxiety and depression, respectively, (20). The results are consistent with our study. The results of multivariate logistic regression analysis in this study showed that participants with low BMI and low family income were found to be more likely to have depression., and participants with a less than high school level of education were more likely to experience anxiety. According to extant literature (2, 4), this result may be related to several factors. First, The diagnosis of NTM disease is a difficulty in the prevention and treatment of tuberculosis, and the clinical manifestations of NTM lung disease are similar to tuberculosis, so the diagnosis of NTM needs to meet the clinical and microbial standards (21). However, the sputum culture results of most patients do not meet the diagnostic standards, which leads to the delay in the diagnosis of NTM patients, and thus leads to the long time for patients to see a doctor and delayed treatment. No appropriate medication, resulting in anxiety and depression during treatment. Second, Since most NTMS are resistant to commonly used anti-mycobacterium drugs, it is difficult to treat NTM disease, and most of the curative effects are not ideal (22). The commonly used anti-NTM disease drug treatment plan has a long cycle and many adverse drug reactions, resulting in increased treatment costs and high treatment cost loads (23). Patients with limited financial means experience certain economic burdens. While opting for treatments, they are also concerned about the treatment cost, which ultimately leads to depression. Third, NTM disease is more common in people with lean body size; this may be related to factors such as the varied secretion levels of leptin (24). However, research on the association between BMI and emotions remains limited. At the same time, NTM is a chronic wasting disease. The worse the nutritional status of patients, the slower the recovery from the disease, which could also lead to depression in patients. In addition, participants with a less than high school level of education were more likely to experience anxiety. This can be attributed to the different levels of education, patients in social relationships for a long time, psychological and physical aspects, low cultural level, development, efficacy, and poor awareness of NTM, as well as limited access to information channels. Therefore, In clinical nursing work, for nursing staff, guiding patients to retain qualified specimens correctly can help find pathogenic bacteria and symptomatic medication. Including the method of sputum, the choice of mouthwash, pay attention to the sterility of the container, are the content we need to educate. During the patient’s treatment, nurses must talk to the patient actively and patiently, inform the patient of the importance of nutrition, nutritional supplementation can be given as early as possible in patients at nutritional risk and always pay attention to the adverse reactions of the patient’s medication, liver damage, nervous and mental system, blood system, kidney damage, etc. (25). Simultaneously, targeted disease guidance and health education, timely answers to questions raised by patients and their families, and early detection of patients with depression and anxiety are crucial to provide patients with more humane care and solve practical difficulties for patients. In addition, nursing staff can teach patients self-help psychological intervention strategies, such as music therapy, to reduce anxiety and depression (Figure 2).

In conclusion, for most of the risk factors affecting the occurrence of anxiety and depression in NTM patients, clinical can be through professional nursing measures, improving the long-term mental health of patients.

The limitations of this study are that the NTM disease was not subdivided into categories, such as cavities, non-cavity lesions, or different types of strains. In the future, subgroup analysis can be conducted through larger sample size studies to assess the psychological status of patients with different types of NTM disease more accurately. In addition, as a preliminary screening and auxiliary means, psychological self-rating scale has its significance, but its main shortcoming lies in its strong subjectivity, easy to be affected by various subjective factors, and its accuracy and reliability may be limited, could may lead to a bias between perceived versus actual symptomatology. Finally, in our study, marital status had no significant effect on the results of the analysis. However, it is reported in the literature that marriage has a protective effect on mental health (26), Men benefit more from marital mental health than women. (27). After consulting the data (27, 28), we need more data on sociodemographic characteristics, socioeconomic characteristics, behavioral factors, and social support to examine the relationship between marriage and anxiety and depression.

## 5. Conclusion

Depression and anxiety are more prominent in patients with non-tuberculosis mycobacteria than in the general population, which deserves further research. Nursing staff work toward identifying anxiety and depression in time and intervene as soon as possible.

## Data availability statement

The raw data supporting the conclusions of this article will be made available by the authors, without undue reservation.

## Ethics statement

The studies involving human participants were reviewed and approved by Ethics Committee of Zhongshan Hospital, Fudan University. The patients/participants provided their written informed consent to participate in this study.

## Author contributions

SN wrote the manuscript. YC collected the data. All authors contributed to the article and approved the submitted version.

## Funding

This work was supported by the Clinical Research Plan of SHDC, Grant No. SHDC2020CR2031B.

## Conflict of interest

The authors declare that the research was conducted in the absence of any commercial or financial relationships that could be construed as a potential conflict of interest.

## Publisher’s note

All claims expressed in this article are solely those of the authors and do not necessarily represent those of their affiliated organizations, or those of the publisher, the editors and the reviewers. Any product that may be evaluated in this article, or claim that may be made by its manufacturer, is not guaranteed or endorsed by the publisher.
